# Prognostic Significance of the Red Cell Distribution Width/Albumin Ratio in the Prediction of the Severity of Acute Biliary Pancreatitis: A Preliminary Report

**DOI:** 10.7759/cureus.30183

**Published:** 2022-10-11

**Authors:** Mustafa Donmez, Orhan Ayata

**Affiliations:** 1 General Surgery, Ankara Bilkent City Hospital, Ankara, TUR; 2 General Surgery, Ankara Yildirim Beyazit University, Ankara, TUR

**Keywords:** albumin, red cell distribution width, severe acute pancreatitis, acute biliary pancreatitis, acute pancreatitis

## Abstract

Objective

Acute pancreatitis (AP) is a common inflammatory disease that should be considered in the etiology of patients presenting to the emergency department with abdominal pain. AP manifests with a clinical picture that can lead to organ failure and even death; therefore, early diagnosis and treatment are essential. In this study, we aimed to evaluate the red cell distribution width (RDW)/albumin ratio (RAR), which, we consider, can be used to determine the clinical course of acute biliary pancreatitis (ABP).

Material and method

The study included 166 patients with ABP. The patient's demographic information, blood values at the time of the first presentation to the emergency department, and radiological results were recorded by screening them retrospectively. Using the Atlanta criteria, the cases were classified into mild, moderately severe, and severe AP groups (MAP, MSAP, and SAP, respectively) and compared.

Results

Of the patients, 121 (72.9%) patients had MAP, 40 (24.1%) had MSAP, and five (3%) had SAP. There was no statistically significant difference between the three groups regarding gender and age. The SAP group had significantly higher median values for the length of hospital stay [19 (4-31) days], white blood cell (WBC) count [20.4 x10^9^/L (9.1-23.3 x10^9^/L)], and creatinine (Cre) [1.4 mg/dL (0.7-3.4 mg/dL)] (p<0.001, p=0.003, and p=0.014, respectively). The RDW and albumin values of all the groups were within normal ranges. RAR was higher in the SAP group but did not statistically significantly different between the groups. In the receiver operating characteristic (ROC) analysis of RAR, the area under the curve (AUC) value was determined as 0.75, sensitivity as 80%, specificity as 70.2%, and positive likelihood ratio as 2.1 (p=0.05).

Conclusion

It is considered that RAR may be a helpful method in determining the course of ABP attacks, but there is a need for studies with a larger series, including all pancreatitis cases.

## Introduction

Acute pancreatitis (AP) is an inflammatory disease of the pancreas characterized by the sudden onset of abdominal pain. Among the essential factors in the etiology of AP are gallstones and alcohol consumption [1-4]. The clinical picture varies from a mild pancreatitis attack to much more severe outcomes, such as organ failure and even death [5-7]. Studies have shown that in patients with AP attacks, the probability of developing complications is around 30%, and the death rate can reach 10% [1,2,7]. Therefore, it is important to identify cases that may develop complications or cause death in the early period and manage their treatment accordingly. For this purpose, in addition to various biochemical tests, various scoring systems, including the Ranson criteria, Acute Physiology and Chronic Health Evaluation II (APACHE II), Balthazar score, and Glasgow score, have been used. However, the clinical applicability of these methods is limited due to their time-consuming, complex nature and low economic feasibility [1-3,7].

In the current study, we aimed to evaluate the effectiveness of the red cell distribution width/albumin ratio (RAR) in determining the severity of acute biliary pancreatitis (ABP). Since both parameters in this ratio are included in routine blood tests, this evaluation would not create an additional economic burden. It can potentially help identify cases that will develop complications in the early period by producing results in a short time. To our knowledge, this is the first study on this subject in the literature.

## Materials and methods

The study included patients who presented to the emergency department of Ankara City Hospital between February 2019 and July 2022, were diagnosed with ABP as a result of evaluation and were admitted to the general surgery ward for treatment. For this reason, patients under 18 years and those with non-biliary pancreatitis, malnutrition or nutritional problems, malignancy, chronic liver and kidney disorders, anemia, or those receiving treatment were excluded from the study. The study was approved by Ethics Committee 1 of Ankara City Hospital (decision number: E1-22-2844) and conducted by the tenets of the Declaration of Helsinki.

The patient's demographic information (age and gender), date of the first admission due to ABP, date of discharge, results of blood tests performed at the time of the first presentation to the emergency department [whole blood count, C-reactive protein (CRP), glucose, creatinine (Cre), blood urea nitrogen (BUN), amylase, lipase, albumin, alanine aminotransferase (ALT), and aspartate aminotransferase (AST), RAR] and radiological imaging findings [ultrasound (US), computed tomography (CT), and magnetic resonance imaging (MRI)] were retrospectively obtained by screening the electronic database of the hospital.

The diagnosis of AP was made according to the revised Atlanta criteria [8] based on the presence of at least two of the following conditions: 1) sudden onset, persistent, severe, radiating epigastric pain in the back, 2) serum amylase or lipase level greater than three times the normal upper limit, or 3) characteristic findings of pancreatitis on contrast-enhanced CT, MRI, or the US. In addition, considering the same criteria, the cases were classified into three groups mild (no local or systemic complications or organ failure), moderately severe (local or systemic complications or transient organ failure lasting less than 48 hours), and severe (persistent organ failure lasting more than 48 hours) AP (MAP, MSAP, and SAP, respectively).

Statistical analysis

Statistical analyses were performed using SPSS version 20.0 (SPSS, Inc, Chicago, IL). Data were analyzed with the Chi-square test, Kruskal-Wallis test, Analysis of Variance (ANOVA), and the receiver operating characteristic (ROC) curve analysis as appropriate. Normally distributed data were expressed as mean±SD, while data that did not show normal distribution were presented as median with range. The results were presented as frequency (n), percent (%), mean ± standard deviation (SD), and median (minimum and maximum range) values. A p-value of less than 0.05 was considered statistically significant.

## Results

Of the 309 patients, 166 were included in the study after evaluating recurrent hospitalizations and applying exclusion criteria. The number of female patients was 102 (61.4%), and that of male patients was 64 (38.6%). The mean age of the patients was 56.8 ± 18.7 years. Of the patients, 121 (72.9%) had MAP, 40 (24.1%) had MSAP, and five (3%) had SAP. There was no statistically significant difference between the three groups regarding gender and age (p = 0.117 and p = 0.436, respectively). In the evaluation of hospitalization duration, the median length of hospital stay was statistically significantly longer in the SAP group [19 (4-31) days] (p < 0.001).

According to the evaluation of the laboratory results, the median WBC count of the whole sample was 11.3 x10^9^/L (3.9-23.9 x10^9^/L), which was above the normal limit (3.9-10.2 x10^9^/L). The WBC count of the SAP group was 20.4 x10^9^/L (9.1-23.3 x10^9^/L), which was statistically significantly higher than the remaining two groups (p = 0.003). The median CRP value of the whole sample was 10mg/L (0-337 mg/L), above the normal range (0-5 mg/L). The median CRP value was the highest in the SAP group [20.5 mg/L (0-26.1 mg/L)], but there was no statistically significant difference between the groups (p = 0.423). The median Cre value of all the patients was within the normal limits [0.8 mg/dL (0.4-3.4 mg/dL), reference: 0.7-1.3 mg/dL]. The median Cre value of the SAP group was 1.4 mg/dL (0.7-3.4 mg/dL), which was statistically significantly higher than the remaining groups (p = 0.014).

When albumin was evaluated, the mean albumin values were within the normal limits (32-48 g/L) in all three groups, but the SAP group had a statistically significantly lower albumin value (39.2 ± 5.9 g/L) (p = 0.009). The mean RDW value of the whole sample was within normal limits (14.1 ± 1.2%, reference: 11.5-16%), and there was no statistically significant difference between the groups (p = 0.212). The whole sample's median calcium value was 9.2 mg/dL, indicating a normal level (8.7-10.4 mg/dL). However, in the SAP group, the median calcium value was significantly lower [8.4 mg/dL (7.7-9.8 mg/dL)] compared to the remaining two groups (p = 0.039). The mean RAR was determined as 0.33 ± 0.04 in the MAP group, 0.35 ± 0.07 in the MSAP group, and 0.39 ± 0.07 in the SAP group. Although RAR was higher in the SAP group, the difference was close but did not reach statistical significance (p = 0.056) (Table 1).

**Table 1:** Characteristics of patients MAP: mild acute pancreatitis, MSAP: moderately severe acute pancreatitis, SAP: severe acute pancreatitis, WBC: white blood cell, RDW: red cell distribution width, SD: standard deviation, CRP: C-reactive protein, RAR: red cell distribution width/albumin

	MAP (n = 121)	MSAP (n = 40)	SAP (n = 5)	p
Gender (n, %)				0.117
Female Male	80 (66.1%)	20 (50%)	2 (40%)	
	41 (33.9%)	20 (50%)	3 (60%)	
Age (median, range)	59 (20-92)	59.5 (19-88)	66 (49-86)	0.436
Length of hospital stay (median, range)	4 (1-25)	7.5 (3-44)	19 (4-31)	< 0.001
WBC count (median, range)	10.8 (4.0-23.9)	14.4 (3.9-23.7)	20.4 (9.1-23.3)	0.003
RDW (mean ± SD)	14.1 ± 1.2	14.1 ± 1.1	15.0 ± 1.2	0.212
CRP (median, range)	10 (0-337)	20 (0-220)	20.5 (0-26.1)	0.423
Creatinine (median, range)	0.8 (0.4-1.4)	0.9 (0.4-1.6)	1.4 (0.7-3.4)	0.014
Albumin (mean ± SD)	42.8 ± 4.0	40.7 ± 4.9	39.2 ± 5.9	0.009
Calcium (median, range)	9.2 (7.9-11)	9.1 (6.5-10.1)	8.4 (7.7-9.8)	0.039
RAR (mean ± SD)	0.33 ± 0.04	0.35 ± 0.07	0.39 ± 0.07	0.056

**Figure 1:**
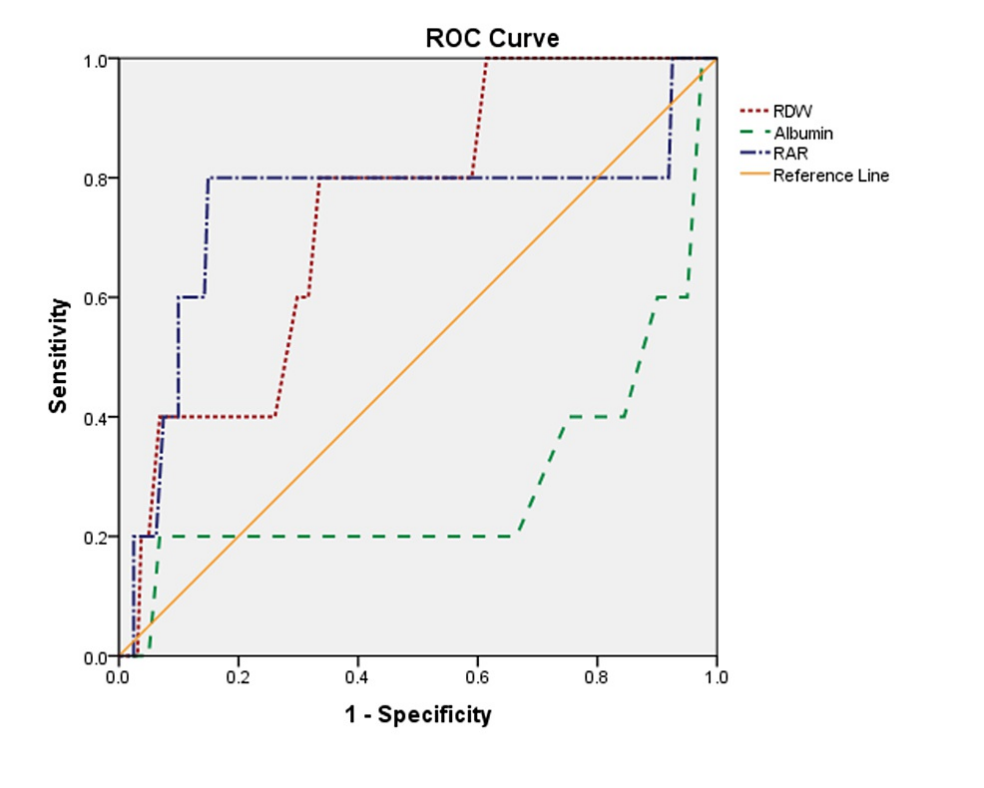
ROC curves for RDW, albumin, and RAR

The ROC analysis was performed for the red cell distribution width (RDW), albumin, and RAR. For RDW, the cut-off value was 14.3, the area under the curve (AUC) was 0.74, the sensitivity was 80%, and the specificity was 61.5%. The cut-off, AUC, sensitivity, and specificity values of albumin were determined as 47.5, 0.29, 20%, and 91.3%, respectively. Lastly, RAR had a cut-off value of 0.34, an AUC value of 0.75, a sensitivity of 80%, and a specificity of 70.2% (Table 2, Figure 1).

**Table 2:** ROC curve analyses of RDW, albumin, and RAR ROC: receiver operating characteristic, RDW: red cell distribution width, RAR: red cell distribution width/albumin, AUC: area under the curve, LR+: positive likelihood ratio

Variable	Cut-off	AUC	Sensitivity	Specificity	LR+	p
RDW	14.3	0.74	80%	61.5%	2.0	0.07
Albumin	47.5	0.29	20%	91.3%	2.3	0.11
RAR	0.34	0.75	80%	70.2%	2.1	0.05

**Figure 2:**
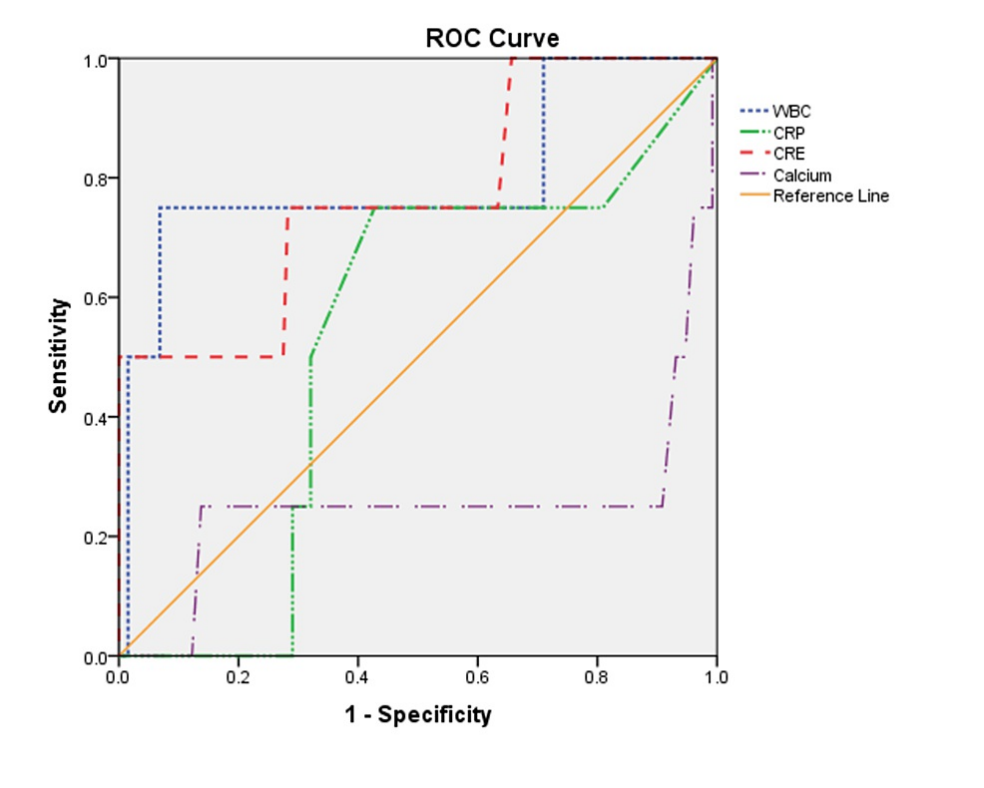
ROC curves for WBC count, CRP, Cre, and calcium WBC: white blood cell, CRP: C-reactive protein, Cre: creatinine

The ROC analysis was also performed for the WBC count, CRP, Cre, and calcium parameters. Accordingly, the cut-off, AUC, sensitivity, and specificity values were determined as 13.7, 0.79, 60%, and 70%, respectively for WBC count; 18, 0.53, 75%, and 57.3%, respectively for CRP; 0.9, 0.77, 80%, and 60.9%, respectively for Cre ; and 9.8, 0.25, 20%, and 88.2%, respectively for calcium (Table 3, Figure 2).

**Table 3:** ROC curve analyses of WBC count, CRP, Cre, and calcium WBC: white blood cell, CRP: C-reactive protein, AUC: area under the curve, LR+: positive likelihood ratio

Variable	Cut-off	AUC	Sensitivity	Specificity	LR+	p
WBC count	13.7	0.79	60%	70%	2.0	0.04
CRP	18	0.53	75%	57.3%	1.8	0.85
Creatinine	0.9	0.77	80%	60.9%	2.0	0.16
Calcium	9.8	0.25	20%	88.2%	1.7	0.9

## Discussion

AP is a severe inflammatory disease that affects pancreatic tissue and can cause multi-organ failure and even death. Although most AP attacks are mild and can resolve with short-term treatment, some cases require more aggressive treatment protocols. Identifying these patients in advance will assist clinicians in appropriately managing patients and reducing morbidity and mortality levels [1,2,7,9]. In the current study, the rate of mild pancreatitis cases was higher. These patients represented the group where organ failure did not develop, and local or systemic complications were not observed. As stated in a study presenting the revised Atlanta criteria [8], this group of patients can usually be discharged from the hospital in the early stage of AP. In our study, the MAP group's median length of hospital stay was determined as 4 (1-25) days, which is consistent with the previously reported duration.

Studies have shown that as the severity of AP increases, the prognosis worsens, and the mortality rate increases to approximately 50% in severe pancreatitis. In addition, it has been suggested that advancing age also affects the increase in the mortality rate [1,3,5,6,8,9]. Although there was no statistically significant difference in our study, the median age of the SAP group was higher [66 (49-86) years] compared to the remaining two groups. One (20%) patient that had an SAP attack died.

To date, many evaluation systems have been used to determine the prognosis of AP. This disease can have important consequences in managing its treatment better and thus reducing mortality. Among these, the Ranson criteria are perhaps the most commonly used method. However, the disadvantage of this method is that it takes 48 hours to produce results to guide decisions. Another scoring system is the APACHE II system, developed mainly for evaluating patients in intensive care units. However, this system includes many additional examinations and parameters that help determine the prognosis of AP. Therefore it does not seem to be a practical and economical method [1-3,6,7].

AP is essentially an inflammatory event. In order to identify an inexpensive, fast, and reliable method to determine the prognosis of patients, previous studies have investigated parameters such as WBC, CRP, Cre, albumin, and RDW, which are expected to change in blood in the presence of inflammation. However, conflicting results have been presented concerning the efficacy of these methods [1,2,4-7,9]. In our study, the median WBC and CRP values in all the groups were above the normal range. However, the median creatinine level was significantly higher in the SAP group compared to the normal values observed in the remaining groups. All the patient groups found the mean albumin level to be within normal limits.

RDW is a parameter used to determine the variability of the size of peripheral circulating erythrocytes and is automatically measured in the whole blood test. It is mainly used in the differential diagnosis of anemia. However, RDW has also been used in many studies to determine the clinical course in many disease groups, including cardiovascular, lung, gastrointestinal, and malignancies [2,6,10-12]. There are also different opinions on this subject. For example, one study concluded that RDW was not useful in determining the clinical course of AP [5]. In contrast, another study suggested that this parameter effectively determines the prognosis of gastrointestinal system diseases, especially AP [11]. In the current study, all three groups' mean RDW values were within normal limits, and no statistically significant difference was found.

Recently, many studies have been conducted with the idea that RAR may provide better results in determining the prognosis of diseases compared to the individual evaluation of either parameter in this ratio [13-15]. We conducted the current study for the same purpose, and to our knowledge, our study is the first to determine the predictive ability of RAR in the prognosis of ABP. Our results support the idea mentioned above. Yoo et al. [16] reported that RAR was more effective than the RDW level in predicting the mortality rate in patients with acute respiratory distress syndrome. Similarly, two different studies have reported that a high RAR value is associated with increased mortality in patients with diabetic ketoacidosis and cancer [17,18]. In our study, although RAR was not statistically significantly different between the groups, it was higher in patients with an SAP attack. When we further evaluated RDW, albumin, and RAR with the ROC analysis, we determined that the results of RDW and RAR were close to each other, but the AUC value of the latter was higher. We attributed the slight difference to the much lower number of people in the SAP group than in the remaining two groups, and we consider that the effectiveness of RAR will be more evident as the number of patients with SAP increases.

Our study had certain limitations, and it had a retrospective and single-center design and was conducted with a relatively small number of patients. To better evaluate the effectiveness of RAR, there is a need for multicenter studies that will include a higher number of patients and all pancreatitis cases.

## Conclusions

In this study, the effectiveness of RAR was investigated, seeking a fast, reliable, and economical method to determine the severity of AP. Consistent with our hypothesis, the RDW and albumin levels were within normal values in all three groups when examined individually. It was observed that they did not contribute to the clinical course of these cases. However, although the number of patients with SAP was relatively low, it was concluded that RAR might be an appropriate method for identifying these patients. Since this is the first study on this subject, there is a need for further studies with wider participation covering all pancreatitis cases to confirm our findings.

## References

[1] Zhang W, Hu J, Yao B, Yang X, Song L, Yin T, Liang L (2017). Evaluation of early prognostic factors of mortality in patients with acute pancreatitis: a retrospective study. Gastroenterol Res Pract.

[2] Wang D, Yang J, Zhang J, Zhang S, Wang B, Wang R, Liu M (2015). Red cell distribution width predicts deaths in patients with acute pancreatitis. J Res Med Sci.

[3] Shi L, Zhang D, Zhang J (2020). Albumin-bilirubin score is associated with in-hospital mortality in critically ill patients with acute pancreatitis. Eur J Gastroenterol Hepatol.

[4] Yarkaç A, Kose A, Bozkurt Babuş S, Ates F, Orekici Temel G, Ölmez A (2019). The value of hematological parameters in acute pancreatitis. Ulus Travma Acil Cerrahi Derg.

[5] Yılmaz EM, Kandemir A (2018). Significance of red blood cell distribution width and C-reactive protein/albumin levels in predicting prognosis of acute pancreatitis. Ulus Travma Acil Cerrahi Derg.

[6] Yao J, Lv G (2014). Association between red cell distribution width and acute pancreatitis: a cross-sectional study. BMJ Open.

[7] Liu G, Tao J, Zhu Z, Wang W (2019). The early prognostic value of inflammatory markers in patients with acute pancreatitis. Clin Res Hepatol Gastroenterol.

[8] Banks PA, Bollen TL, Dervenis C (2013). Classification of acute pancreatitis--2012: revision of the Atlanta classification and definitions by international consensus. Gut.

[9] Li Y, Zhao Y, Feng L, Guo R (2017). Comparison of the prognostic values of inflammation markers in patients with acute pancreatitis: a retrospective cohort study. BMJ Open.

[10] Ren Q, Liu H, Wang Y, Dai D, Tian Z, Jiao G, Liu X (2021). The role of red blood cell distribution width in the severity and prognosis of community-acquired pneumonia. Can Respir J.

[11] Goyal H, Lippi G, Gjymishka A, John B, Chhabra R, May E (2017). Prognostic significance of red blood cell distribution width in gastrointestinal disorders. World J Gastroenterol.

[12] Meynaar IA, Knook AH, Coolen S (2013). Red cell distribution width as predictor for mortality in critically ill patients. Neth J Med.

[13] Long J, Xie X, Xu D (2021). Association between red blood cell distribution width-to-albumin ratio and prognosis of patients with aortic aneurysms. Int J Gen Med.

[14] Seo YJ, Yu J, Park JY (2022). Red cell distribution width/albumin ratio and 90-day mortality after burn surgery. Burns Trauma.

[15] Hong J, Hu X, Liu W (2022). Impact of red cell distribution width and red cell distribution width/albumin ratio on all-cause mortality in patients with type 2 diabetes and foot ulcers: a retrospective cohort study. Cardiovasc Diabetol.

[16] Yoo JW, Ju S, Lee SJ, Cho YJ, Lee JD, Kim HC (2020). Red cell distribution width/albumin ratio is associated with 60-day mortality in patients with acute respiratory distress syndrome. Infect Dis (Lond).

[17] Lu C, Long J, Liu H, Xie X, Xu D, Fang X, Zhu Y (2022). Red blood cell distribution width-to-albumin ratio is associated with all-cause mortality in cancer patients. J Clin Lab Anal.

[18] Zhou D, Wang J, Li X (2021). The red blood cell distribution width-albumin ratio was a potential prognostic biomarker for diabetic ketoacidosis. Int J Gen Med.

